# Estimating Dormant and Active Hematopoietic Stem Cell Kinetics through Extensive Modeling of Bromodeoxyuridine Label-Retaining Cell Dynamics

**DOI:** 10.1371/journal.pone.0006972

**Published:** 2009-09-22

**Authors:** Richard C. van der Wath, Anne Wilson, Elisa Laurenti, Andreas Trumpp, Pietro Liò

**Affiliations:** 1 Computer Laboratory, University of Cambridge, Cambridge, United Kingdom; 2 Ludwig Institute for Cancer Research Ltd., Lausanne Branch, University of Lausanne, Epalinges, Switzerland; 3 Division of Cell Biology, Deutches Krebforschungzentrum (DKFZ), Division of Cell Biology, DKFZ-ZMBH Alliance, Heidelberg, Germany; 4 Heidelberg Institute for Stem Cell Technology and Experimental Medicine (HI-STEM), Heidelberg, Germany; Harvard Medical School, United States of America

## Abstract

Bone marrow hematopoietic stem cells (HSCs) are responsible for both lifelong daily maintenance of all blood cells and for repair after cell loss. Until recently the cellular mechanisms by which HSCs accomplish these two very different tasks remained an open question. Biological evidence has now been found for the existence of two related mouse HSC populations. First, a dormant HSC (d-HSC) population which harbors the highest self-renewal potential of all blood cells but is only induced into active self-renewal in response to hematopoietic stress. And second, an active HSC (a-HSC) subset that by and large produces the progenitors and mature cells required for maintenance of day-to-day hematopoiesis. Here we present computational analyses further supporting the d-HSC concept through extensive modeling of experimental DNA label-retaining cell (LRC) data. Our conclusion that the presence of a slowly dividing subpopulation of HSCs is the most likely explanation (amongst the various possible causes including stochastic cellular variation) of the observed long term Bromodeoxyuridine (BrdU) retention, is confirmed by the deterministic and stochastic models presented here. Moreover, modeling both HSC BrdU uptake and dilution in three stages and careful treatment of the BrdU detection sensitivity permitted improved estimates of HSC turnover rates. This analysis predicts that d-HSCs cycle about once every 149–193 days and a-HSCs about once every 28–36 days. We further predict that, using LRC assays, a 75%–92.5% purification of d-HSCs can be achieved after 59–130 days of chase. Interestingly, the d-HSC proportion is now estimated to be around 30–45% of total HSCs - more than twice that of our previous estimate.

## Introduction

Multi-potent stem cells are required to regenerate self-renewing tissues such as the skin, gut, and hematopoietic system. They have the capacity to provide both life-long self-renewal and to generate all the terminally differentiated cell types of each lineage. In order to protect against oncogenic mutations, most immature adult stem cells are thought to divide infrequently and be predominantly in a quiescent state (reviewed in [1]). In addition, quiescence has been postulated to prevent stem cell exhaustion. Bone marrow (BM) hematopoietic stem cells (HSCs) are crucial to maintain lifelong production of all blood cells. Due to the technological advances provided by flow cytometry, the existence of multiple monoclonal antibodies directed to stem cell specific cell surface antigens, and in vitro and in vivo assays that can quantitate their functional capacity, mouse BM HSCs are amongst the most well characterized (both phenotypically and functionally) adult stem cells. All functional activity resides within the Lin
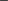
Sca1
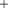
cKit
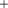
CD150
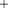
CD48
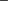
CD34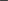 population (hereafter termed HSCs) that comprises around 0.001% of mouse BM. Although HSCs have been shown to be predominantly in a transient resting state of cell cycle and are therefore thought to divide infrequently, it has always been assumed that this is a stochastic process with the entire HSC pool turning over every few weeks. Indeed, the earliest studies estimated the doubling time of individual HSCs to be between 17.8 and 30 days with the entire HSC pool turning over every 57 days. Moreover, these studies excluded the existence of a dormant HSC population. Thus the common dogma was that despite their relative transient quiescence, all HSCs nevertheless regularly entered and exited the cell cycle.

**Figure 1:**
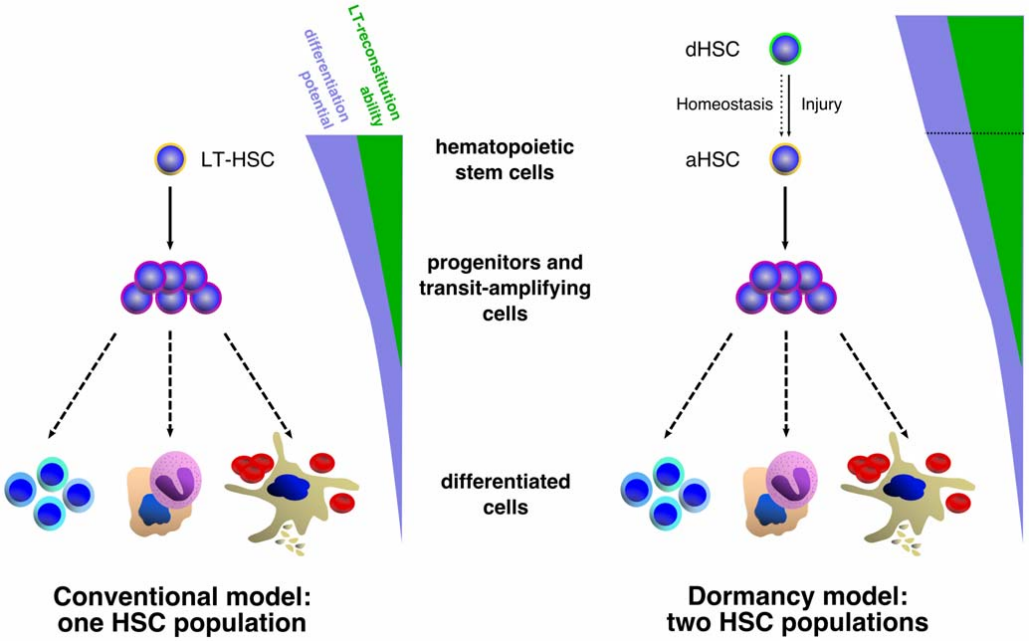
Conventional vs. dormant population HSC hierarchy. The hierarchical organization of the hematopoietic system has long been recognized, with rarely-dividing multipotential HSCs producing rapidly dividing lineage-restricted transit-amplifying and committed progenitors which in turn will give rise to all differentiated cell types of the blood. Within the HSC population, two possible models can be envisaged. In the conventional model (left panel), the HSC population is homogeneous with respect to cell cycle entry with the entire HSC pool turning over every few weeks. In contrast, in the dormant HSC model (right panel), the hierarchical organization of the hematopoietic system includes the phenotypic HSC pool, in which two subpopulations can be defined based on their relative turn-over frequencies. An active HSC (a-HSC) population is responsible for the day-to-day maintenance of the hematopoietic system, while a second population, the dormant HSC pool (d-HSC), cycles only a few times over the life span of the mouse in a homeostatic situation (dashed arrow) but is activated and participates in replenishment of the hematopoietic system after injury (solid arrow).

In recent studies however, we and others have identified a population of dormant mouse HSCs (d-HSCs) within the HSC BM population that divides only about 5 times in the life span of a mouse [2], [3]. We combined flow cytometry with Bromodeoxyuridine (BrdU) and histone-2B-GFP (H2B-GFP) label-retaining assays that depend on the ability of dormant HSCs to retain a DNA or nuclear protein label over a long chase period (up to 306 days). Cycling cells will however rapidly lose this type of label as it is diluted by half after every complete cell cycle, and is undetectable by flow cytometric assays after 4 to 5 cell divisions. We further showed that while d-HSCs possess most of the multi-lineage long-term self-renewal activity, they are efficiently activated in response to BM injury. After re-establishment of homeostasis, activated HSCs return to dormancy, suggesting that HSCs are not stochastically entering the cell cycle but reversibly switch from dormancy to self-renewal under conditions of hematopoietic stress (Figure 1, right panel).

While putative dormant stem cell populations have been observed in situ in both the skin and the intestine using the classical BrdU-label retaining cell assay [4], their precise phenotypic characterization has been elusive due to the lack of specific surface markers and functional assays for these organs. Recently, studies using the H2B-GFP transgenic system described in [2] under the control of the K5 epidermal specific promoter have provided functional evidence for an epidermal stem cell with limited cycling potential [5]. Nevertheless, neither of these stem cell populations is as well characterized as the HSC particularly in the context of quiescence or reversible activation after injury. Thus, to date the existence of a bona fide d-HSC remains confined to the hematopoietic system.

Here we present deterministic and stochastic computational models of our BrdU label-retaining cell (LRC) data. We show that those models that assume the existence of a slowly cycling subpopulation and heterogeneity over time are able to describe the observed experimental data the most satisfactorily. The LRC models we define here are more extensive than those we used in [2] in that we now also model BrdU uptake and refine the way in which the model accounts for the BrdU detection threshold (BDT). It turns out that there exist a multiple nonlinear dependency between the number of divisions during uptake, the number of divisions during chase, and the BDT. By carefully elucidating this relationship and incorporating the results in our model we were able to better estimate HSC turnover rates. We further describe a stochastic model that enabled us to simulate BrdU-based LRC assays as a Markov Process. Results from these stochastic simulations shows the observed long term BrdU retention cannot be explained by stochastic variation alone. More importantly, the two-sample Kolmogorov-Smirnov test indicates that the experimental data are more likely a sample from a heterogenous population of cells than a homogeneous population.

## Results

### BrdU uptake influences parameter estimates during chase

We have previously defined a mathematical model of BrdU LRC data to support the dormant HSC hypothesis [2]. There our approach was a simple comparison between two versions of the model to see whether the observed BrdU labeling data can be more satisfactorily described by a one-population model or by a two-population model. In addition to HSC proliferation kinetics, the BDT was estimated since it can have a confounding effect on the observed LRC data. Even though BrdU labeling assays are characterized by two phases, the shorter uptake or pulse phase (BrdU present for 10–13 days) and a longer chase phase (BrdU absent for up to 306 days), our focus was previously exclusively on BrdU dilution data. The reason was due to the fact that the discriminative power for both heterogeneity in HSC proliferation parameters and the BDT can be expected to lie in the chase phase of the data only. LRCs can, by definition, only be observed during the chase phase when labeling is diluted. In addition, the only information about the BDT we can learn from the uptake data is that 50% labeled DNA (thus after one or more uptake divisions) is an upper bound for our detection threshold estimate. If BrdU detection by flow cytometry was less sensitive than 50%, cells would need to divide at least twice before we could detect them as BrdU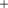, making BrdU labeling very inefficient. Nevertheless the BDT upper bound is apparent in the chase phase data where a detection threshold of more than 50% would imply rapid loss of labeling, similar to what would be the case if chromosomes segregated asymmetrically [6], [7].

Here our models in [2] are elaborated upon and extended with the aim to improve model parameter estimates. We have included equations for BrdU uptake and refined the way we account for the fluorescent detection threshold of BrdU. Although BrdU uptake data has little discriminative power when investigating HSC heterogeneity, actual parameter estimates are in fact strongly influenced when they are based on both BrdU uptake and chase data, as opposed to chase data only (which was the case in [2]). This observation is based on the concept that cells that have divided more in the presence of BrdU need more divisions to dilute the BrdU they have taken up. The BDT further complicates matters since some cells will take 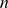 chase divisions to transcend the detection threshold whilst others that have divided more during uptake will take 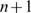 divisions. However, for some other BDT these same cells might take an equal number of divisions to dilute their label. This multiple nonlinear relationship is summarized in Table 1 where we can clearly see that at a threshold of 5%, 1 and 2 uptake division cells will lose label after 4 divisions, and 3 and more uptake division cells will lose label after 5 divisions. At a 7.5% threshold, 1 uptake division cells will lose label after 3 divisions whilst all the others (2 or more uptake divisions) will take 4 divisions. Interestingly all cells will lose label after 3 divisions at a 12.5% threshold. It is thus obvious that a complex interplay between the number of divisions during uptake, the BDT, and the number of divisions during chase exists. Any BrdU model used to infer cell division kinetics should carefully address these three factors. See Methods for details on how the values in Table 1 were computed.

**Table 1:** BrdU detection threshold.

BDT	% DNA strands labeled	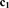	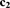	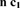	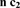
1	1.25%	1,2	3,4,…	6	7
2	2.5%	1,2	3,4, …	5	6
3	3.75%	1	2,3, …	4	5
4	5%	1,2	3,4, …	4	5
5	6.25%		1,2, …		4
6	7.5%	1	2,3, …	3	4
7	8.75%	1	2,3, …	3	4
8	10%	1,2	3,4, …	3	4
9	11.25%	1,2,3	4,5, …	3	4
10	12.5%		1,2, …		3

BDT: BrdU detection threshold (minimum number of labeled DNA strands that can be detected). For each BDT we divide cells into two groups during the chase phase (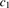 and 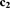) depending on how many times a cell has divided during BrdU uptake. We then give 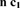 and 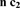, the number of times cells in each group has to divide to go below the detection threshold. Consider a BDT of 4 strands for example: cells that have divided once or twice during uptake will dilute their BrdU in 4 divisions, whilst it will take 5 divisions for cells that have divided 3 or more times during uptake. This table was completed using Figure 6.

### Long term LRC data indicate a slowly dividing subpopulation

**Figure 2:**
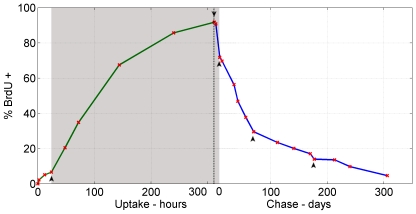
Observed experimental data of BrdU uptake and chase. Green: uptake; blue: chase; red x: mean observed data; vertical dotted line: time at which BrdU was removed. Each time point represents between 5 and 11 mice. The dose of BrdU administered is 180 mg i.p. per mouse at the start followed by water containing 800 micrograms per ml BrdU continuously for 10–13 days (for more details on the experimental procedure see [2]). Plotting BrdU uptake on a timescale of hours and chase on a timescale of days clearly shows a markable change in kinetic slope at 5 time points (black arrows). Definitive biological events can be attributed to the first three changes, which motivated a three-stage (second stage shaded in solid grey) parameter estimation strategy, as discussed in the main text. The change occurring at chase day 70 (fourth arrow) can be regarded as the starting point of the long label retaining tail in the graph. The observed data at chase day 177 (fifth arrow) seems like an outlier since subsequent time points return to the kinetic slope as observed prior to day 177.

The empirical BrdU LRC data we obtained are shown in Figure 2, with the uptake data on a timescale of hours for improved readability. The novelty of this dataset is the exceptionally long chase period, as previous BrdU datasets only included uptake [8] or tracked label dilution no longer than 70 days [9]–[11] or 120 days [6] at most. Strikingly the rate of label dilution in Figure 2 decreases after chase day 70 flattening out as label is retained on the long term. In this section we investigate possible causes of the observed long term BrdU retention and conclude that it must be due to a slowly dividing subpopulation of cells.

Long term retention of experimentally labeled DNA in cells such as observed in Figure 2 are either an artefact of the experimental procedure used or can be explained by some biological property of the cells under scrutiny. Our quantitative approach accounts for two possible experimental confounding factors that might create an illusion of label retention. The first is controlling for the BDT, which as highlighted above, is an absolute necessity for more accurate parameter estimation. Apart from its influence on parameter estimation the BDT also provides a possible explanation for label retention, since if BrdU detection by flow cytometry was extremely sensitive, cells would still be detected as BrdU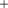 even after many chase divisions and hence it will appear as if cells have long term BrdU retention. Alternatively it might be possible that the natural stochastic variation between mice at the HSC level is much larger than expected. In this case the observed long tail in Figure 2 may be due to an inadequate or unintentional skew sample of mice examined at each time point. To investigate this possibility we predicted confidence intervals using variance estimates of a stochastic version of our model discussed in the following section.

In terms of biological properties of stem cells explaining label retention two independent hypotheses have been postulated. Firstly it has been proposed in the “Immortal Strand Hypothesis” that stem cells divide frequently, but in doing so asymmetrically recognize and retain the “old” labeled mother DNA strand, while the newly synthesized unlabeled chromosomes are selectively distributed to the non stem cell daughter [12], [13]. Alternatively stem cells with a very slow division rate during chase will retain DNA label much longer than those that divide more frequently. Recently we, and others have shown that label retention, which cannot be convincingly explained by an asymmetric segregation mechanism, has also been observed in nuclear protein labeling assays, such as that using a H2B-GFP fusion protein under control of specific promoters [2], [3]. Moreover the “Immortal Strand Hypothesis” has recently been seriously challenged for HSCs [6], [14], [15], although it has been found that the LRC data of [6] provides insufficient information about the segregation mechanism of chromosomes, but nevertheless supports HSC heterogeneity including a dormant population [7]. The models we present here have therefore assumed random chromosome segregation so that the presence or absence of dormancy is the only biological hypothesis we have to address. Similar to our approach in [2] two versions of the LRC model were thus implemented. First a one-population version that models the data as being observed from a single population of cells with homogeneous turnover rates was defined. Secondly this model was extended to a two-population version that assumes heterogenous turnover rates between the two subpopulations.

Each model is defined by a set of Ordinary Differential Equations (ODEs) that describes the rate of change of the proportion of labeled cells over time (see Methods for details). The dynamics of the model equations are determined by the self-renewal (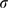), differentiation (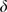) and death (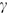) rates of the cell population in question. These parameters were assumed to be constant and constrained to maintain a steady state. By this assumption we thus effectively estimated the average turnover rates over particular periods in time. If HSC dynamics are constant, as during homeostasis, this poses no problem and serves as a convenient description of the net effect of state-dependent (i.e. non constant) parameters. However careful study of the observed LRC data in Figure 2 reveals 5 points in time where a peculiar change in kinetic slope is apparent, indicated with black arrows. There is an initial lag phase after BrdU is first added to the system. After 24 hours a sudden increase in BrdU uptake rate can be observed (first arrow), which extends until 312 hours (13 days) when BrdU is removed (second arrow). At this time point, the cells immediately start to dilute their label as they continue to divide and hence the percentage of BrdU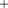 cells rapidly decreases, until day 10 of chase (third arrow) when it seems that the BrdU dilution rate decreases and the curve flattens out. Each one of these three time points we just noted has a biological interpretation. The most obvious is the second when BrdU is removed at 312 hours and the cells immediately start to dilute out the label. The initial lag phase (0–24 h) during uptake followed by the sudden increased incorporation of BrdU after 24 hours of pulse (the first time point) is however much less obvious. As some toxic effects of BrdU on cycling cells have been previously reported [16], [17], a proliferative signal is most likely induced in HSCs (commencing around 24 h) in response to peripheral injury caused by BrdU. Indeed, we have confirmed that this is the most likely reason for the changes in proliferation kinetics at this point [2]. Importantly, this proliferative burst is the most likely explanation as to why dormant HSCs can be efficiently labeled with BrdU (around 90% after 13 days) in the first place. Similarly, when BrdU is removed at 312 hours we first observe a rapid loss of BrdU label over about 10 days before HSC proliferation returns to “normal” rates. This initial rapid loss of label is also a consequence of the toxic effects of BrdU on the periphery, as HSCs are still cycling in response to injury signals mediated by the presence of BrdU. Once the BrdU is removed, it may take several days for the injury status to resolve and for homeostasis to be re-established.

Unlike the first 3 time points discussed above, there is no known biological event that can be attributed to the change occurring at day 70. However, the change of kinetic slope at this time point can be regarded as the starting point of the label retaining tail we observed. Since we have implemented measures to control for the observed tail, we are satisfied that the model can readily account for the alteration in kinetics of BrdU loss at day 70. At day 177 an unexpected drop in BrdU retention can be observed. This is most likely due to non-specific external influences on the mice (such as a mild infection) during the long chase period, which activated the d-HSCs. The change of slope at chase day 177 is different from the previous four in that it is a solitary change with the subsequent time points returning to the kinetic slope as observed prior to day 177. Thus the observed data at chase day 177 could be regarded as outlying, particularly as omitting this time point had negligible effects on parameter estimation (data not shown).

From the discussion above we thus motivate parameter estimation in three regions, rather than one set of parameter estimates for all time points. Our three-stage parameter estimation strategy proceeded as follows:

1. Starting with 0% of the cells labeled, we fitted parameters to uptake data observed in the first 24 hours. These parameters are an estimate of the homeostatic proliferation rates, but we kept in mind that little information can be conveyed by the only three observed data points (excluding day 0), hence these estimates carry less weight than the homeostatic estimates of stage 3.
2. Continuing with the distribution predicted by stage 1 as the initial condition, we estimated a new set of parameters for the rest of the uptake data and the first 10 days of chase (shaded in grey in Figure 2). Since we hypothesized d-HSC to be activated in this stage we expected an increase in parameter estimates (“hematopoietic stress” proliferation rates). In this stage there is a switch between the BrdU uptake and chase phases. Since the BDT has a profound effect during chase the cells have to be partitioned into two groups at the onset of chase - those that lose labeling after 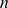 divisions and those that lose labeling after 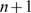 divisions. See the discussion in the previous section for more details.
3. Finally we estimated parameters for the rest of the chase data, from day 10 onwards. The initial labeling proportions amongst the two partitions are obtained from the day 10 predictions in stage 2 so that we maintain parameter integrity between the three different estimate regions. In this region we assumed that HSC turnover has settled back to homeostatic rates, hence dormant cells, if present, would have switched back to a dormant state.

**Figure 3:**
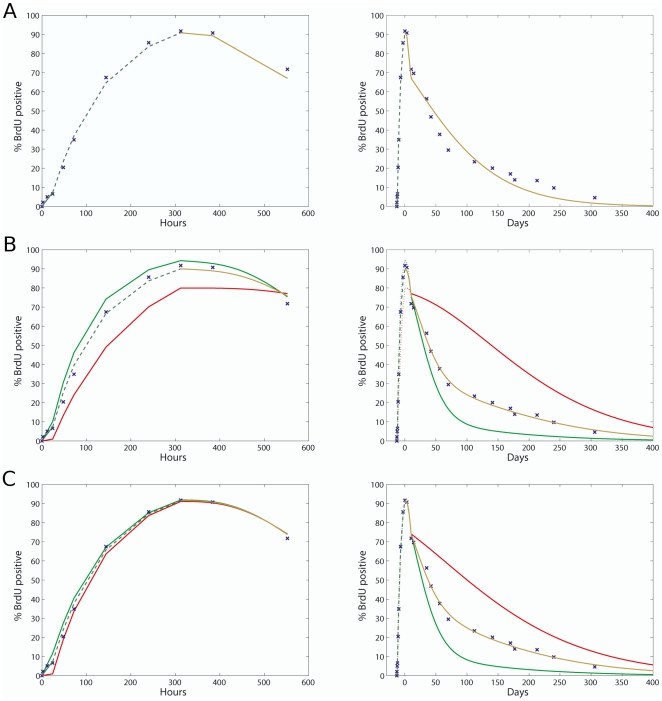
Deterministic LRC model predictions of BrdU content. Brown line: HSC chase; red line: d-HSC; green line: a-HSC; dashed line: HSC uptake; blue x: observed data. Left panel: stage 1 & 2 predictions (uptake and first 10 days of chase) on a timescale of hours; right panel: uptake and chase predictions on a timescale of days. (A) One-population model predictions. This model can satisfactorily describe BrdU uptake but not the long term label-retention. (B) Two-population model predictions with a BDT of 4 and 30% d-HSC proportion. The effect of a smaller d-HSC population is visible in the left panel of this plot. (C) Two-population model predictions with a BDT of 6 or 7 and 40% d-HSC proportion. This model gave the best overall goodness-of-fit. Activation of the d-HSCs can be clearly seen in the left panel where both d-HSC and a-HSC are predicted to take BrdU up at the same rate (cycling about once every 10 days). During chase d-HSCs return to a dormant state and are predicted to divide about once every 165 days, whilst a-HSCs divide once every 31 days, diluting label much faster than the d-HSCs.

The predictions of our LRC models after all parameters have been optimized are shown in Figure 3. Here we plot the results of the best one-population model (A), and two different parameter settings for the two-population model (B and C) together with the d-HSC (red) and active HSC (a-HSC) (green) BrdU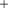 percentages. On the right the full model predictions are shown on a time scale of days and on the left only stage 1 and 2 (uptake and first 10 days of chase) on a time scale of hours. Table 2 summarizes the best performing parameter sets for each of the one-population and two-population models based on the Residual Sum of Squares (RSS) measure. From these results it is clear that the one-population model does not fit the observed data nearly as well as the two-population model whose best RSS value of 71.7 is more than five times lower that the best one-population RSS value of 391.9. Considering each parameter estimation stage individually however, we see that both model versions fit the observed data during stages 1 and 2 satisfactorily. This is to be expected since the d-HSCs have not been activated in stage 1 so we are mostly observing the a-HSCs, allowing the one-population model to have a good fit. Conversely we start observing the d-HSCs after 24 hours (stage 2) since they have now been activated, but since they are dividing at an increased rate (most likely close to the a-HSC rate) it still appears as if there is a homogenous rate of BrdU uptake. In stage 3 the d-HSCs have switched back to a resting state, but unlike in stage 1 we do observe them since most of the d-HSCs have been labeled in stage 2. Thus in stage 3 there is heterogeneity in the BrdU dilution rate causing the one-population model to completely fail in describing the data. In contrast the two-population model describes the stage 3 data extremely well. The fact that the one-population model can successfully describe the uptake data but not the chase data is in agreement with our analysis earlier that the discriminative power for heterogeneity only lies in the chase data.

**Table 2:** Goodness-of-fit results for the LRC model.

BDT	DP	RSS	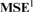	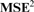	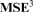	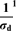	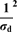	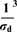	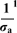	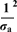	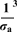
9	0%	391.9	1.54	6.56	27.54				26.1	10.4	95.5
3	30%	131.8	1.51	11.7	4.31	208.3	16.2	122.2	18.7	7.2	25
4	30%	113.8	1.51	9.42	3.94	208.3	15	134.4	18.7	7.6	26.5
5	30%	87	1.51	5.79	3.22	208.3	13.8	150.2	18.7	8.2	32.3
6 & 7	30%	80.6	1.51	4.07	3.6	208.3	11	189.9	18.7	9.3	35.6
6 & 7	35%	73.6	1.5	3.97	3.25	208.3	10.2	170.3	17.5	9.6	32.4
**6 & 7**	**40%**	**71.7**	**1.5**	**3.89**	**3.22**	**208.3**	**9.9**	**165.1**	**16.1**	**9.8**	**30.5**
6 & 7	45%	73.7	1.5	3.8	3.41	208.3	9.8	149.1	14.8	9.8	28
8	35%	95.2	1.5	3.85	4.93	208.3	10.2	193.9	17.5	10.2	40.3

BDT: BrdU detection threshold, DP: Dormant proportion, RSS: Residual sum of squares, MSE: Mean Squared Error, superscripts indicate modeling stage 1,2 or 3. DP of 0% indicates the one-population model. Self-renewal rates 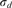 and 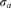 are inverted to units of days.

**Figure 4:**
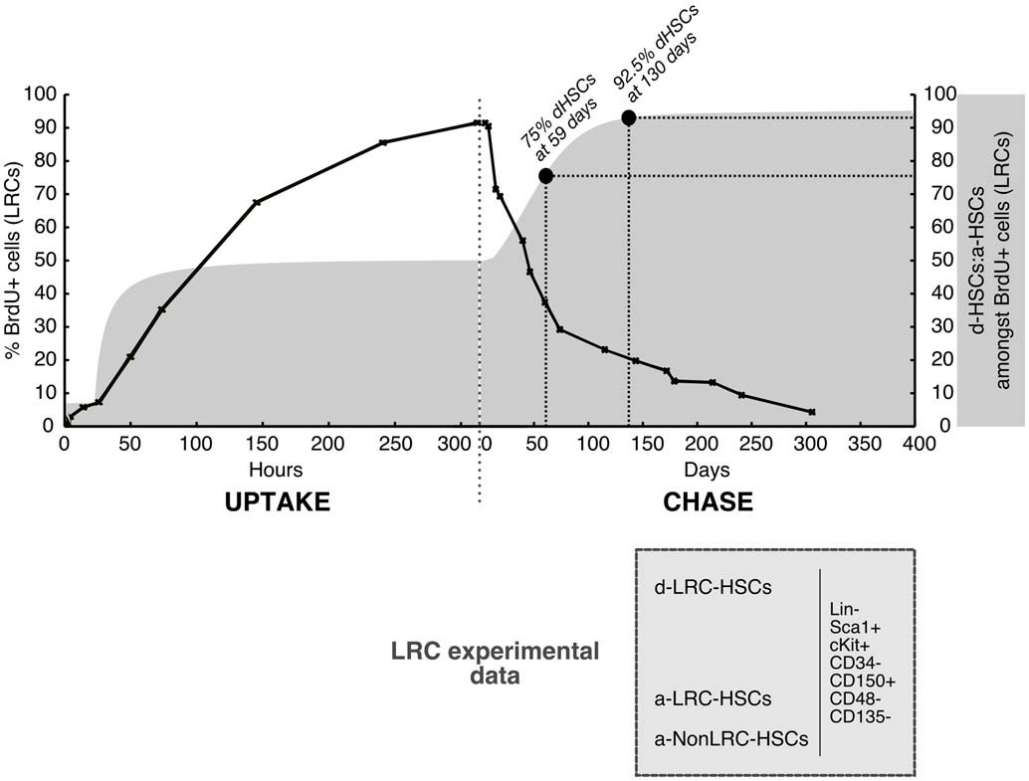
Summary of experimental data and modeling conclusions. Kinetics of uptake and loss of BrdU within the phenotypic HSCs (Lin
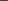
Sca1
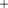
CD34
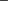
CD150
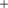
CD48
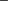
CD135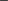) as determined experimentally (solid black line) overlaid with the relative proportion of d-HSCs (dormant HSC) and a-HSCs (active HSCs) amongst BrdU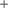 phenotypic HSCs estimated by our modeling (dark grey shaded curve). The populations that can be found at any time point of a LRC experiment amongst phenotypic HSCs are indicated in the light grey box. The time points of chase at which the d-HSCs represent 75% and 92.5% of the LRC phenotypic HSCs are indicated by solid black circles.

The best two-population model (highlighted in bold in Table 2) suggests a BDT of 6 or 7 DNA strands, which both map to the same 3–4 division partitioning (Table 1). Not only does this parameter set result in the lowest RSS, its Mean Squared Error (MSE) for stage 2 and 3 is also consistent. Moreover, the stage 2 estimate for the d-HSC self-renewal rate 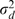 and a-HSC self-renewal rate 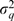 are similar - supporting the idea that d-HSCs are activated to self-renew at the a-HSC rate during stage 2. Indeed all d-HSC self-renewal estimates in Table 2 clearly predict a *resting-activated-resting* scheme for stages 1, 2 and 3 respectively, indicating that activation of d-HSCs is reversible. Using our estimated parameters we predicted the ratio of d-HSCs to a-HSCs amongst the BrdU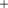 cells (shaded area of Figure 4). This information is of great interest for biologists who want to isolate d-HSCs during an LRC experiment. Our model predicts that 75%–92.5% purification can be achieved if BrdU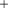 cells are isolated between chase day 59 and 130.

In summary our parameter estimates (based on a maximum RSS of 80.6) are: BDT: 6–7 strands (3–4 divisions); d-HSC proportion: 30–45%; d-HSC self-renew during stage 3 (putative homeostatic rate): once every 149–193 days; a-HSC self-renew during stage 3: once every 28–36 days.

### Stochastic variation cannot explain long term label retention

The ODE-based LRC models described above result in deterministic solutions, and hence describe average population dynamics. It is widely accepted that stochasticity is an inherent property of biological systems and modeling them as such is an area of great interest [18]–[20]. In spite of being deterministic our ODE-based models have been very useful and efficient for parameter estimation. However, we have already highlighted the need - under the d-HSC hypothesis - to rule out stochastic variability as a cause of the observed long-term label retention. Describing individual cells as agents and keeping track of the labeling status of their chromosomes proved to be a simple and useful stochastic model of BrdU data [7]. Its discrete properties made this approach especially effective in implementing the BrdU detection threshold by alleviating the need for continuous approximation and grouping of the number of labeled DNA strands in a cell. However the relationship between the predictions of our deterministic ODE model and a stochastic agent-based model for the same set of parameters is unclear. Any inference of stochastic variance estimated by an agent-based model using parameters optimized by our ODE model are thus somewhat troublesome.

**Figure 5:**
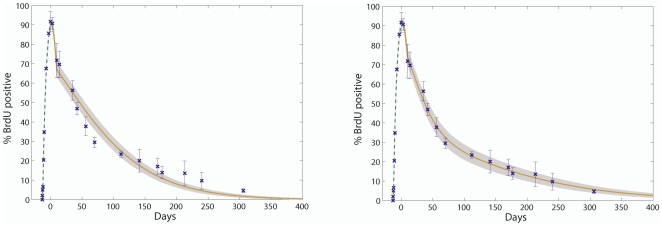
Stochastic LRC model predictions of BrdU content. Mean observed data and variation are indicated in blue; shaded area represents estimated variance of the predictions from 1000 stochastic simulations, each with and initial 3750 HSCs. Left plot is one-population stochastic predictions. Right plot is two-population stochastic prediction corresponding to the deterministic model of Figure 3C with a BDT of 6 or 7 strands. It is clear that the one-population predictions and hence stochastic variation alone cannot explain the observed long-term label retention. The two-population predictions in turn encapsulates all observed averages, although there are still some unexplained variation at some time points.

Fortunately all reaction rates of the ODE model are first order, which means we can derive a Markov Process whose average behavior is exactly described by the ODE model for the same set of parameters [20]. Assuming that the system we model adheres to the Markov property (cells don't have memory of their labeling states and number of cells in the past), and that times between events are exponentially distributed, descriptive statistics of the system can be calculated from multiple stochastic simulations with the reassurance that the deterministic predictions will be correctly described by the mean stochastic trajectory. For each set of equations in our ODE model we derived a Master Equation that defines the transition kernel of a continuous time state-discrete Markov Process (see Methods for details). Using the best parameter sets deduced with our ODE models, multiple simulations for each of the one and two-population models enabled us to estimate confidence intervals of predictions (shaded areas of Figure 5). If the confidence interval of the one-population model encapsulates the late state data then it is quite possible to observe long-term label retention without HSC dormancy. Clearly this is not the case in Figure 5. In contrast at least all the mean experimental data falls inside the two-population confidence interval. There are however, a few time points where the observed variability is larger than predicted. This is most likely either due to fewer mice examined at those particular time points or additional variation not accounted for by our model, like different number of initial HSCs. However, little variation has been observed at chase day 306, which happens to be encapsulated by the two-population confidence interval. Moreover the two-sample Kolmogorov-Smirnov test [21] suggests the observed data is more likely to be a sample from the two-population model predictions (null hypothesis not rejected, p-value = 0.133) than the one-population model predictions (null hypothesis rejected, p-value = 0.0314).

Taken together both the deterministic and stochastic models thus strongly support a dormant subpopulation in HSCs.

## Discussion

While it has been long accepted that HSCs are quiescent, meaning they rarely divide, our previous work has demonstrated heterogeneity among the phenotypic long term HSC population (Lin
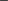
Sca1
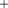
CD117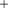 CD34
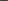
CD150
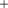
CD48
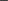
CD135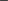), with the existence of a dormant subset of cells (d-HSCs), which proliferates only several times during their lifespan, and an active subset which is responsible for day-to-day maintenance of the hematopoietic system [2]. Nevertheless, upon different stimuli such as 5-FU, G-CSF or IFNa [2], [22], these d-HSCs can exit dormancy and proliferate to the same rate as their active counterparts. As most current chemotherapeutic strategies target actively cycling cells, dormant and oncogenically mutated stem cells would be immune to such treatment, and could potentially cause leukemic relapse unless they can be pharmacologically stimulated to enter an active state. Hence the challenge is to understand which drugs should be combined with which chemotherapeutic agents in order to eradicate even the most primitive cancer stem cells (reviewed in [23]). The BrdU compound used in our LRC experiments, is one of such substances that can make d-HSCs exit dormancy. Therefore useful information can be gathered by carefully analyzing the kinetics of BrdU uptake and dilution.

Here we have refined our previous mathematical modeling and thereby identified some biologically relevant parameters. First, our new modeling approach (see below) estimates the percentage of d-HSCs amongst Lin
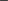
Sca1
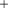
CD117
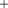
CD34
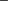
CD150
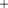
CD48
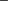
CD135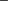 (phenotypic HSCs) to be around 40% (bold row in Table 2) under homeostatic conditions, markedly higher than our previous evaluation. It is important to distinguish this parameter from the relative proportion of d-HSCs amongst BrdU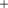 LRCs at any point during the LRC experiment (plotted in solid light grey in Figure 4). This latter number is of practical interest because it predicts that after 130 days of chase the a-HSC contribution to the label retaining pool (BrdU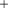 stem cells) is negligible as d-HSCs would comprise more than 90% of the remaining BrdU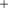 LRCs at this time point (Figure 4). Notably, the curve asymptotes at 95%, meaning that a totally pure population of d-HSCs can never be purified by label-retaining assays alone, underlining the need to search for other d-HSC markers. Indeed, future biological studies will be focused on elucidating new surface markers that could be used to isolate d-HSCs without the need to perform long-term label retaining experiments. Once identified, putative d-HSC-specific markers may be utilized to screen tumors for potential cancer stem cells.

The proportion of d-HSCs amongst phenotypic HSCs (regardless of their label-retaining state) would also change throughout the LRC experiment. At time 0 (when the mice are first exposed to BrdU, and prior to peripheral injury signals being registered by HSCs) the homeostatic situation would prevail, therefore around 40% of HSCs would be d-HSCs. However, during the injury phase (after 24 h of exposure to BrdU and up until removal of BrdU at 10 days) the proportion of d-HSCs amongst all HSCs would decrease until close to zero. After cessation of BrdU, the 40% homeostatic plateau would be gradually regained.

One paradoxical question that invariably comes to mind when trying to prove dormancy in cells using BrdU-based LRC assays is: if in what we observe there are dormant cells, and hence these dormant cells are all labeled during the pulse period, these labeled dormant cells can no longer be considered as dormant since only cells that have cycled, thus non-dormant cells, can be labeled in the first place? Our data showing that BrdU can indirectly induce activation of HSCs, provides an elegant explanation for this phenomenon [2] which additionally raises the possibility that the d-HSCs can reversibly switch between active and dormant states (Figure 1). A further issue is whether BrdU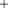 HSCs undergo cell cycle arrest during chase due to the incorporated BrdU, thereby leading to the appearance of a slow cycling subset. Although we cannot completely exclude this possibility, there are two reasons why we think this is not the case. The first is that we [2], and others [3] have confirmed our observations of the same slowly cycling HSC subset using a second, non-chromosomal model (a nuclear protein, Histone2B-GFP). Secondly, our mathematical models predict that, at the onset of chase, half of the BrdU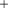 cells are a-HSCs (see Figure 4) which indeed have ‘normal’ rates of cycling (28–36 days) similar to what was previously estimated for the entire HSC pool. Thus it is unlikely that BrdU creates a slow-cycling population.

In the LRC model, when homogeneous parameter values are assumed for the entire dataset, optimization simply collapses - it is impossible to fit both the uptake and chase profiles with a homogeneous turnover rate, even with a heterogenous cell population. We thus adopted a three-stage parameter estimation strategy making sure each stage is based on careful biological motivations. Since the LRC dataset at our disposal is the most detailed to date, and due to our thorough and novel treatment of the correlation between the BDT and number of uptake divisions (Table 1), we were able to estimate parameters very accurately using our three-stage strategy. This is evident in the good fit (Figure 3) and low RSS values (Table 2) we were able to achieve. Our estimates for the d-HSC and a-HSC division rates are nevertheless in agreement with the chase only model estimates in [2], albeit that we found a larger range of values to fit the data. It is notable that the a-HSC division rate of 28–36 days is similar to previous HSC division rate estimates where a homogeneous HSC population was assumed [8]. Most interesting is the d-HSC proportion prediction of 30–45% - markedly higher than the 15% we estimated in [2]. A clear pattern that emerged during parameter optimization was that larger d-HSC proportions tend to fit uptake data (stage 2) better. The fact that we did not consider uptake data in [2] thus serves as a possible explanation as to why we had a lower estimate for the d-HSC proportion.

One striking observation that we can infer from the LRC modeling is that it takes about 10 days for d-HSCs to regain dormancy after BrdU withdrawal. Interestingly, this recovery period corresponds to that estimated by transcriptional profiling of HSCs after a single 5-FU injection [24], suggesting that the mechanism controlling return to dormancy might be independent of which stimulus led to activation.

All our findings above were supported by stochastic modeling which ruled out cellular level variation as a cause for observing long-term label retention. Our Master Equation approach conveniently allowed us to estimate variation for a set of parameters whilst the average random walk remained true to the deterministic prediction. Future work will focus on exploring additional unexplained variation on a genetic or molecular level. One major challenge will be how to deal with the unavoidable increase in number of unknown parameters.

It is not clear from the LRC models what the impact of the d-HSC population would be on hematopoietic regulation. The LRC equations defined here, although modeling proportion of labeled cells rather than actual cell numbers, implicitly define cell population dynamics. However, this implicit HSC model cannot explain dynamic homeostasis since all the kinetic parameters (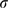, 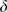, 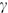) are independent of the population state. An interesting question that arises is whether d-HSC hematopoiesis (Figure 1, right panel) is evolutionarily superior to the previously widely accepted dormant-free hematopoiesis (Figure 1, left panel). This question is extremely hard to answer with human reasoning alone but current work focuses on a computational treatment of the problem.

In summary, whilst we previously [2] presented biological evidence supporting the concept of a d-HSC population, this current work provides additional support for our d-HSC hypothesis from a Computational Biology perspective. We have shown that both deterministic and stochastic models of observed BrdU labeling dynamics strongly support heterogeneity in the BM HSC population with a small slowly cycling portion of cells (d-HSCs) responsible for long term label retention. Parameter estimation indicated that at least a third of the HSC population are d-HSCs that divide about once every 149–193 days with a-HSCs dividing once every 28–36 days. We further predict that more than 90% purification of d-HSCs can be achieved after 130 days of chase using LRC assays. In this study we have focused on the modeling of LRC-based HSC kinetic data rather than the modeling of dynamic maintenance and restoration of homeostasis in the hematopoietic system. The major motivation for not using a dynamic model for parameter estimation is the inherent data scarcity of the LRC results. We thus defined the LRC model as simple as possible to limit the number of parameters that need to be estimated. As such the trade-off between increased model complexity and a small improvement in the goodness-of-fit did not validate the use of models with more than 2 populations. Moreover, the large difference between the turnover estimates for d-HSCs and a-HSCs suggest that the transition distribution between a dormant and active state is indeed disjunct and bimodal rather than continuous.

## Methods

### Mapping BrdU intensity to number of divisions

**Figure 6:**
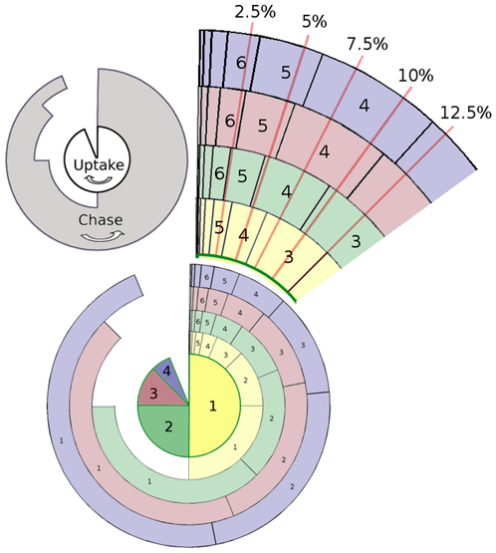
Average theoretical BrdU percentage of a single cell for a given number of divisions during uptake and chase. Yellow: cell has divided once during uptake; green: cell has divided twice during uptake; red: cell has divided thrice during uptake; violet: cell has divided four times during uptake, more than four divisions will also be in this group; red lines: detection thresholds. Slices of each uptake pie are cumulative. Uptake divisions follow in a clockwise direction and chase divisions follow in an anticlockwise direction. Slices intersected by red lines indicate the number of divisions for the cell to fall below the detection threshold.

The nonlinear mapping of BrdU intensity to the number of divisions during uptake and chase are summarized in Table 1, whose values are computed from Figure 6. Figure 6 depicts the average real-valued labeling percentage of a single cell based on up to 4 uptake divisions, and the decrease in label corresponding to each of the 1 to 4 uptake divisions. Also shown is a slice in more detail where 5 different detection thresholds are indicated with red lines. The proportions in Figure 6 can be calculated when realizing that half of a cell's DNA is newly synthesized after each mitotic cell division. During BrdU uptake all newly synthesized DNA will be BrdU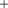 so that 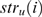, the average number of labeled DNA strands in a single cell after uptake division 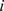 is given by the recursive expression
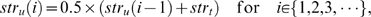
where 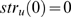 and 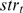 is the total number of DNA strands (twice the number of chromosomes). Here we assume that chromosomes segregate randomly and hence it is possible for chromosomes to have both their DNA strands labeled after two or more uptake divisions. Conversely all newly synthesized DNA during chase is BrdU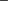 (unlabeled) so that 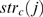, the average number of labeled DNA strands in a single cell after chase division 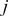 is given by
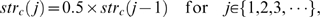
where 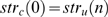 after 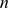 uptake divisions. Note that we are calculating the average number of labeled DNA strands for a single cell and non-integer numbers of strands are thus possible. The biological interpretation must however be made in a cell population context to make sense. For example 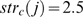 means that 50% of cells in a particular population have 2 labeled DNA strands and the other 50% have 3 labeled DNA strands.

The total number of uptake and chase divisions we have to consider is dependent on the assumed BDT. In theory the smallest change in BrdU intensity is by one nucleotide, which poses a potential dilemma in the number of possible detection thresholds to model. Fortunately BrdU intensity is diluted in units of chromosomes upon cell division so that intensity differences on a nucleotide level can be safely ignored. Moreover, no chromosome can have both its DNA strands labeled after one chase division. We thus know that label intensity will be reduced by multiples of single DNA strands of chromosomes during chase (after the first division) and that the least possible label intensity in a cell is 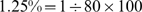 in the case of a mouse. Note that we have assumed that differences in the sizes of chromosomes are negligible.

### Modeling BrdU uptake and chase

We modeled the LRC data of Figure 2 by a system of coupled Ordinary Differential Equations (ODEs) that describe the rate of change of the number of BrdU labeled cells over time. Two versions of this model are defined, a one-population model and a two-population model. The one-population version assumes a single active cell population represented by 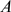. The two-population version in turns assumes an additional dormant cell population (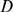) whose differentiated daughter cells enter the 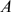 population. Each model has a set of equations for BrdU uptake (indicated by subscript 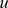) and two sets of equations for the chase phase, subscript 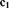 for the group of cells diluting labeling after 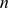 divisions and subscript 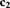 for the group of cells diluting labeling after 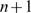 divisions. Dynamics are determined by the rates at which cells self-renew 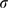, differentiate 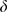, or die 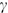. Here we use subscript 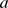 and 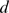 to indicate the different rates of the active and dormant populations respectively. A cell that self-renews has undergone cell cycling and hence is replaced by two daughter cells with changed label intensities. Label intensity is mapped to the number of chase divisions a cell has undergone from a reference point (see Table 1) so that an equation can be defined for cells at each division level. For example, during uptake active cells that have divided 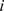 times in the presence of BrdU will be presented by 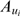, and chase group 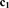 active cells that have divided 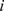 times without BrdU present will be presented by 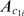. Dormant cell notation follows in a similar fashion. Differentiation happens when a cell changes its phenotype to that of its direct progeny - independent of cell division and hence label intensity is unaffected. Cells that die are removed from the system. The various equations are given below.

### One-population equations



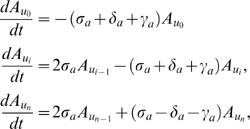
(1)for 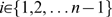.
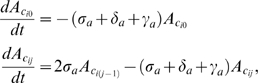
(2)where 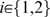 and 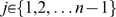 if 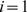, or 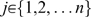 if 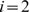.

### Two-population equations



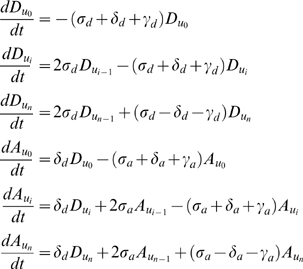
(3)for 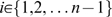.
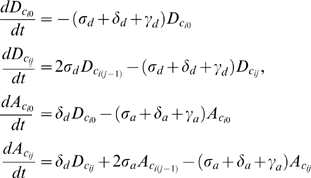
(4)where 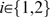 and 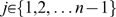 if 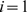, or 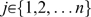 if 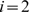.

All the above ODEs are linear and thus have analytic solutions in the form of constrained non-linear multivariate functions (see Supporting Information File S1 for more details). The sets of chase equations are coupled to the uptake equations by partitioning of the uptake cells at chase day 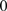 into chase group 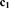 or 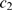 depending on the BDT we model.

We assume that parameters are constrained such that the total cell population remains constant. We use the Residual Sum of Squares 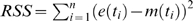, where 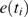 is the experimental value observed at time 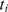 and 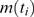 is the predicted value at time 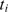, to evaluate goodness-of-fit. Smaller values for RSS indicate a better fit. Parameter values that minimise the RSS of each model were found by using suitable algorithms from the Optimization Toolbox of the Mathworks Matlab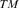 software suite. The Mean Squared Error (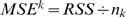) is a normalized measure that can be used to compare the goodness-of-fit during the three stages of parameter optimization where 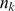 is the number of data points of stage 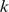.

### Stochastic methods

The LRC model described above can be viewed as a random walk moving through a multi-dimensional hyperspace of cellular species (i.e. all the 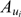, 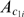, 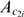, 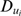, 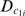, 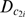 for which we have defined an equation) over time. When the next future state is fully determined by the current state of the system, independent of all previous states, the random walk adheres to the Markov property and is known as a Markov Process. The Markov property is a reasonable assumption for the LRC system we are modeling since, if the current number of cells with their labeling intensities are known, no additional knowledge about the future number of the cells and their labeling states can be gained from earlier cell numbers. We can derive a Master Equation, also known as the Chapman-Kolmogorov equation [25], for each of the one-population and two-population ODE models. The Master Equations can be easily derived if the reactions implied by the ODEs are first written down. All BrdU reactions for a cellular species 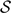 are defined by the general reaction scheme 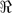,
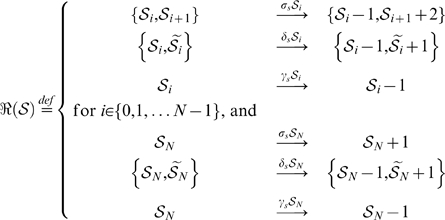
(5)


Here 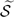 refers to species on the next level of the differentiation hierarchy, thus direct progeny of 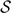. 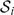 indicate species at division level 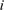 and its interpretation is dependent on the context of 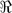, whether it describes BrdU uptake or dilution. For example, in the context of BrdU uptake reactions as described by 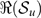, 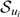 denotes cells that have divided 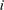 times in the presence of BrdU. Alternatively, for reactions of 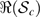 (BrdU chase), 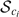 would in turn denote cells that have divided 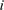 times without BrdU present in their microenvironment - after they have been labelled. Hence there is an inverse context dependent interpretation of 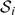 with unlabelled cells during BrdU uptake -and chase indicated by 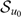 and 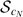 respectively. Note that differentiation happens independent of cell division and hence label intensity is unaffected.

#### One-population Master Equation

The general form of the Master Equation for the one-population models is given by
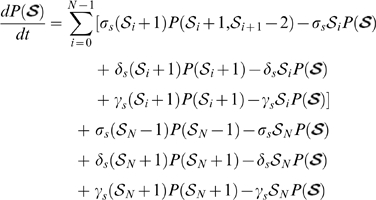
(6)


Where the following equivalences are defined for notational convenience:
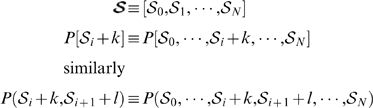



Note that differentiated progeny 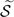 do not appear in the Master Equation since we only model a single population of cells.

We can now describe the BrdU uptake reactions of a homogeneous a-HSC population 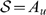 with 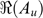, keeping track of 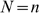 labelling intensities. The Master Equation 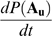 describes the transition kernel of a continuous time Markov Process where we regard 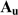 as the state space. For the BrdU chase reactions, 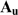 is partitioned into two disjunct groups based on label intensity and BrdU detection threshold. Let 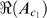 be the reactions for cells that take 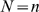 divisions to loose BrdU labelling, and 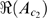 be the reactions for cells that take 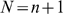 divisions to loose BrdU labelling. We have two state spaces 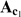 and 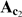, each modelled by the respective Master Equations 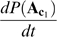 and 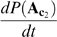.

#### Two-population Master Equation

The general form of the Master Equation for the two-population models is given by
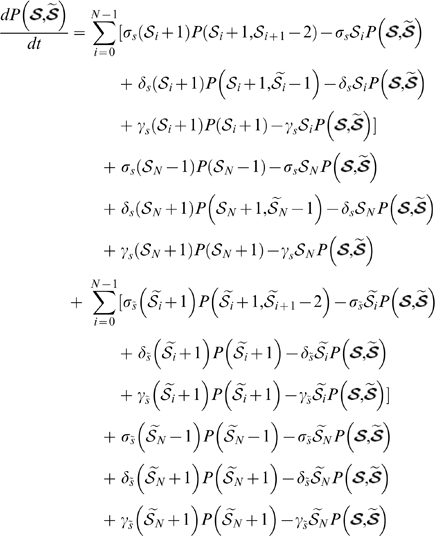
(7)


Again we introduced some equivalences to simplify notation:
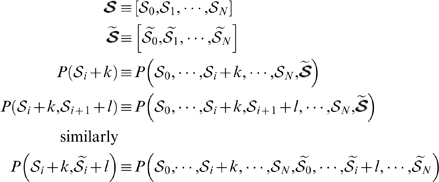



Let 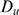 be the d-HSC species during BrdU uptake and let 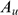 be the a-HSC species during uptake. Also let 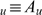. The reactions during BrdU uptake are then given by 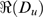 and 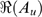, and the Master Equation for the Markov Process by 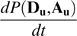.

As before, we have two state spaces when considering BrdU chase, with the partitioning dependent on BrdU detection sensitivity and level of labelling during BrdU uptake. Let 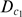 and 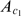 represent the different cell species that falls below the BrdU detection threshold after 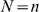 divisions, and let 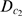 and 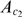 be the ones that looses labelling after 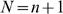 divisions. Reactions for chase in a heterogenous cell population case is then given by 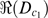, 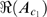, 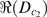 and 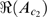. Two Master Equations 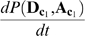 and 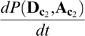 now govern the Markov Processes with state spaces 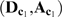 and 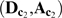 respectively.

The Master Equations defined above have no known analytic solutions but fortunately there are various algorithms available to simulate such processes. In this paper we used one of the most well known, namely the exact Stochastic Simulation Algorithm introduced by Gillespie [26] for simulating biochemical reactions. We performed 1000 simulations, starting each simulation with an initial HSC population of 3750 [1].

#### Two-sample Kolmogorov-Smirnov test

Apart from estimating variances, it was possible to use the two-sample Kolmogorov-Smirnov test [21] to compare the probability distributions of our observed data and simulated data. Our strategy was to randomly sample 137 values (the total number of mice examined in the dataset of Figure 2) from the Markov Process simulations with each sample assigned to one of the time points for which we have observed data. We then performed the two-sample Kolmogorov-Smirnov test on the random sample and experimental data. The whole procedure was repeated 1000 times and the average p-value reported.

### Simulations

Parameter estimates of the LRC model solutions and the two sample Kolmogorov-Smirnov tests were computed using Matlab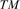. Stochastic simulations were performed with software written in C++. All diagrams were plotted using Matlab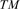 with editing done in the GNU Image Manipulation Program (http://www.gimp.org/) and Inkscape (http://www.inkscape.org/).

## Supporting Information

File S1 Analytic solutions for the LRC model
(0.04 MB PDF)
