# Supplementary material for: Estimating Dormant and Active Hematopoietic Stem Cell Kinetics through Extensive Modeling of Bromodeoxyuridine Label-Retaining Cell Dynamics
Source: PLoS One. 2009 Sep 22;4(9):e6972. doi: 10.1371/journal.pone.0006972 (PMC2745578; doi:10.1371/journal.pone.0006972)
Supplement: File S1 — Analytic solutions for the LRC model (0.04 MB PDF) [file pone.0006972.s001.pdf]

Supporting Information
Estimating Dormant and Active Hematopoietic Stem Cell Kinetics through Extensive Modeling of Bromodeoxyuridine Label-Retaining Cell Dynamics

Analytic solutions for the LRC model

Analytical expression of Equations 1-4 can be derived quite conveniently by writing each system of ODEs in matrix form as

$$\mathbf{x}' = \mathbf{K} \cdot \mathbf{x},$$

where $\mathbf{x}$ is the vector of cell species for which equations are defined in each of the four systems, $\mathbf{x}'$ contains the derivatives of these variables and $\mathbf{K}$ is the coefficient matrix. The solution can be written in analogy to that of first order differential equations

$$\mathbf{x}(t) = \exp(\mathbf{K} \cdot t) \cdot \mathbf{x}(0),$$

where `exp` is the matrix exponential function. The analytical expression of Equations 1-4 only differ in their version of $\mathbf{x}$ and $\mathbf{K}$ and these are given below.

One-population model solution

The expression for $\mathbf{x}$ and $\mathbf{K}$ during uptake is

$$\mathbf{x} = \begin{bmatrix} A_{u_0} \\ A_{u_1} \\ A_{u_2} \\ \vdots \\ A_{u_n} \end{bmatrix} \quad \text{and} \quad \mathbf{K} = \begin{bmatrix} -\Phi & 0 & 0 & \cdots & 0 \\ 2\sigma_a & -\Phi & 0 & \cdots & 0 \\ 0 & 2\sigma_a & -\Phi & \cdots & 0 \\ \vdots & \vdots & \ddots & \ddots & \vdots \\ 0 & 0 & \cdots & 2\sigma_a & (2\sigma_a - \Phi) \end{bmatrix}.$$

During chase, for cells loosing labelling after n divisions, they are

$$\mathbf{x} = \begin{bmatrix} A_{c_{10}} \\ A_{c_{11}} \\ A_{c_{12}} \\ \vdots \\ A_{c_{1(n-1)}} \end{bmatrix} \quad \text{and} \quad \mathbf{K} = \begin{bmatrix} -\Phi & 0 & 0 & \cdots & 0 \\ 2\sigma_a & -\Phi & 0 & \cdots & 0 \\ 0 & 2\sigma_a & -\Phi & \cdots & 0 \\ \vdots & \vdots & \ddots & \ddots & \vdots \\ 0 & 0 & \cdots & 2\sigma_a & -\Phi \end{bmatrix},$$

where $\Phi = \sigma_a + \delta_a + \gamma_a$ in both cases above. The expression for $\mathbf{x}$ and $\mathbf{K}$ during chase, for cells loosing labelling after $n+1$ divisions, follows in a similar fashion.

Two-population model solution

Uptake expressions:

$$\mathbf{x}^\top = \begin{bmatrix} D_{u_0} & D_{u_1} & \cdots & D_{u_n} & A_{u_0} & A_{u_1} & \cdots & A_{u_n} \end{bmatrix}$$

$$\mathbf{K} = \begin{bmatrix} -\Phi_d & 0 & 0 & \cdots & 0 & 0 & 0 & 0 & \cdots & 0 \\ 2\sigma_d & -\Phi_d & 0 & \cdots & 0 & 0 & 0 & 0 & \cdots & 0 \\ 0 & 2\sigma_d & -\Phi_d & \cdots & 0 & 0 & 0 & 0 & \cdots & 0 \\ \vdots & \vdots & \ddots & \ddots & \vdots & \vdots & \vdots & \vdots & \ddots & \vdots \\ 0 & 0 & \cdots & 2\sigma_d & (2\sigma_d - \Phi_d) & 0 & 0 & 0 & \cdots & 0 \\ \hline \delta_d & 0 & 0 & \cdots & 0 & -\Phi_a & 0 & 0 & \cdots & 0 \\ 0 & \delta_d & 0 & \cdots & 0 & 2\sigma_a & -\Phi_a & 0 & \cdots & 0 \\ 0 & 0 & \delta_d & \cdots & 0 & 0 & 2\sigma_a & -\Phi_a & \cdots & 0 \\ \vdots & \vdots & \vdots & \ddots & \vdots & \vdots & \vdots & \ddots & \ddots & \vdots \\ 0 & 0 & 0 & \cdots & \delta_d & 0 & 0 & \cdots & 2\sigma_a & (2\sigma_a - \Phi_a) \end{bmatrix}.$$

Chase expressions, for cells loosing labelling after n divisions:

$$\mathbf{x}^\top = \begin{bmatrix} D_{c_{10}} & D_{c_{11}} & \cdots & D_{c_{1(n-1)}} & A_{c_{10}} & A_{c_{11}} & \cdots & A_{c_{1(n-1)}} \end{bmatrix}$$

$$\mathbf{K} = \begin{bmatrix} -\Phi_d & 0 & 0 & \cdots & 0 & 0 & 0 & 0 & \cdots & 0 \\ 2\sigma_d & -\Phi_d & 0 & \cdots & 0 & 0 & 0 & 0 & \cdots & 0 \\ 0 & 2\sigma_d & -\Phi_d & \cdots & 0 & 0 & 0 & 0 & \cdots & 0 \\ \vdots & \vdots & \ddots & \ddots & \vdots & \vdots & \vdots & \vdots & \ddots & \vdots \\ 0 & 0 & \cdots & 2\sigma_d & -\Phi_d & 0 & 0 & 0 & \cdots & 0 \\ \hline \delta_d & 0 & 0 & \cdots & 0 & -\Phi_a & 0 & 0 & \cdots & 0 \\ 0 & \delta_d & 0 & \cdots & 0 & 2\sigma_a & -\Phi_a & 0 & \cdots & 0 \\ 0 & 0 & \delta_d & \cdots & 0 & 0 & 2\sigma_a & -\Phi_a & \cdots & 0 \\ \vdots & \vdots & \vdots & \ddots & \vdots & \vdots & \vdots & \ddots & \ddots & \vdots \\ 0 & 0 & 0 & \cdots & \delta_d & 0 & 0 & \cdots & 2\sigma_a & -\Phi_a \end{bmatrix}.$$

The expressions for cells loosing labelling after $n+1$ divisions follows in a similar fashion. $\Phi_d = \sigma_d + \delta_d + \gamma_d$ and $\Phi_a = \sigma_a + \delta_a + \gamma_a$ in both cases above.
